# Left ventricular thrombus routine screening with contrast echocardiography in patients with anterior ST-elevation myocardial infarction: is it worth it?

**DOI:** 10.1186/s44348-024-00027-0

**Published:** 2024-08-05

**Authors:** Joana Laranjeira Correia, Gonçalo R. M. Ferreira, João Gouveia Fiuza, Mariana Duarte Almeida, Joana Coelho, Emanuel Correia, José Miguel Correia, Davide Moreira, Nuno Craveiro, Maria Luísa Gonçalves, Vanda Devesa Neto

**Affiliations:** 1 Cardiology Department, Centro Hospitalar Tondela-Viseu, Viseu, Portugal; 2 Internal Medicine Department, Centro Hospitalar Universitário Cova da Beira, Covilhã, Portugal; 3 Universidade da Beira Interior, Covilhã, Portugal

**Keywords:** Anterior wall myocardial infarction, ST elevation myocardial infarction, Thrombus, Echocardiography, Ultrasonographic contrast

## Abstract

**Background:**

Left ventricular (LV) thrombus has a higher incidence among patients with anterior ST-elevation myocardial infarction (STEMI) when compared to other types of acute myocardial infarction and is associated with worse prognosis. The management of LV thrombus diagnosis remains challenging. Contrast echocardiography (transthoracic echocardiography, TTE) has shown potential in improving the accuracy for its diagnosis, thereby influencing treatment strategies concerning antithrombotic/anticoagulation therapy. The aim of this study was to assess the effectiveness of contrast TTE as a routine screening method for detecting LV thrombus in the acute phase of anterior STEMI.

**Methods:**

A prospective, single center, randomized controlled trial was conducted among patients with anterior STEMI. The study group underwent contrast TTE, while the control group received a conventional approach. Demographical, clinical, and diagnostic data were collected. Thrombus detection rates were compared between groups.

**Results:**

A total of 68 patients were included (32 in the study group and 36 in the control group). No substantial baseline differences were observed between groups. Thrombus detection rate was 25.0% in the study group and 13.9% in the control group, however these results did not reach statistical significance (*P* = 0.24). The prevalence of anterior/apical aneurysm was higher in the study group (46.9% vs. 22.2%, *P* = 0.03).

**Conclusions:**

Conventional TTE may be adequate for diagnosing LV thrombus in the acute phase of anterior STEMI; however, further larger-scale and multicenter studies are necessary to obtain more robust and conclusive results. Ultrasound contrast may play a significant role in the detection of anterior/apical aneurysms, which are known risk factors for the subsequent development of thrombus.

**Trial registration:**

NCT06480929 (ClinicalTrials.gov, Retrospectively registered).

**Supplementary Information:**

The online version contains supplementary material available at 10.1186/s44348-024-00027-0.

## Background

The introduction of primary percutaneous coronary intervention (PCI) has resulted in a reduction in mortality rates associated with acute myocardial infarction (AMI). However, postinfarct complications continue to contribute to a worse prognosis. Despite significant advances in the diagnosis and treatment of cardiovascular diseases, the management of left ventricular (LV) thrombus remains challenging [1]. Although its incidence has decreased significantly due to advancements in reperfusion techniques and antithrombotic therapies, the presence of LV thrombus still poses a substantial risk of embolic events [1–3]. The 1-year risk of stroke associated with LV thrombus can reach up to 10% of cases, even with anticoagulation therapy [4].

The development of thrombus following an AMI, particularly in cases of anterior ST-elevation myocardial infarction (STEMI), is influenced by three primary factors: endothelial injury resulting from myocardial infarction, blood stasis caused by LV dysfunction, and hypercoagulability induced by the inflammatory state [1, 2]. Anterior STEMI typically occurs due to the occlusion of the left anterior descending artery and is associated with a worse prognosis compared to other coronary territories, primarily due to the larger area of myocardial supplied [3, 5]. One common complication of anterior STEMI is LV apical akinesia, leading to blood stasis and subsequent thrombus formation. This often occurs within 24 h after AMI and mostly within the first 2 weeks [1–3, 6, 7].

According to the guidelines of the European Society of Cardiology, after a primary PCI, a routine transthoracic echocardiography (TTE) is recommended to assess LV function, evaluate right ventricle and valve function, and also to exclude early postinfarction mechanical complications and LV thrombus [8].

Conventional TTE is easily accessible, safe, and cost-effective. However, this approach is reliant on the operator's skills and could potentially provide inconclusive outcomes or overlook the identification of an intracardiac thrombus, especially in situations involving foreshortened images or small/mural thrombi. Advances in ultrasound technology, including harmonic imaging and the use of contrast agents, have improved the diagnostic accuracy in detecting LV thrombus [9–11].

Ultrasound contrast agents, which are easily accessible diagnostic tools with a favorable cost-effectiveness and safety profile, are used to improve the opacification of the LV and provide clearer delineation of the endocardial borders. This enhancement enables the identification of a distinctive "filling defect," which is a characteristic feature of an intracardiac thrombus [5, 10]. As a result, contrast TTE may significantly impact the diagnosis of LV thrombus, particularly in high-risk patients such as those with anterior-territory STEMI. This has the potential to influence treatment strategies by guiding decisions regarding antithrombotic therapy versus oral anticoagulation [2, 6, 9, 10].

The aim of this study was to assess the effectiveness of contrast TTE as a routine screening method for detecting LV thrombus in the acute phase of anterior STEMI.

## Methods

**Fig. 1:**
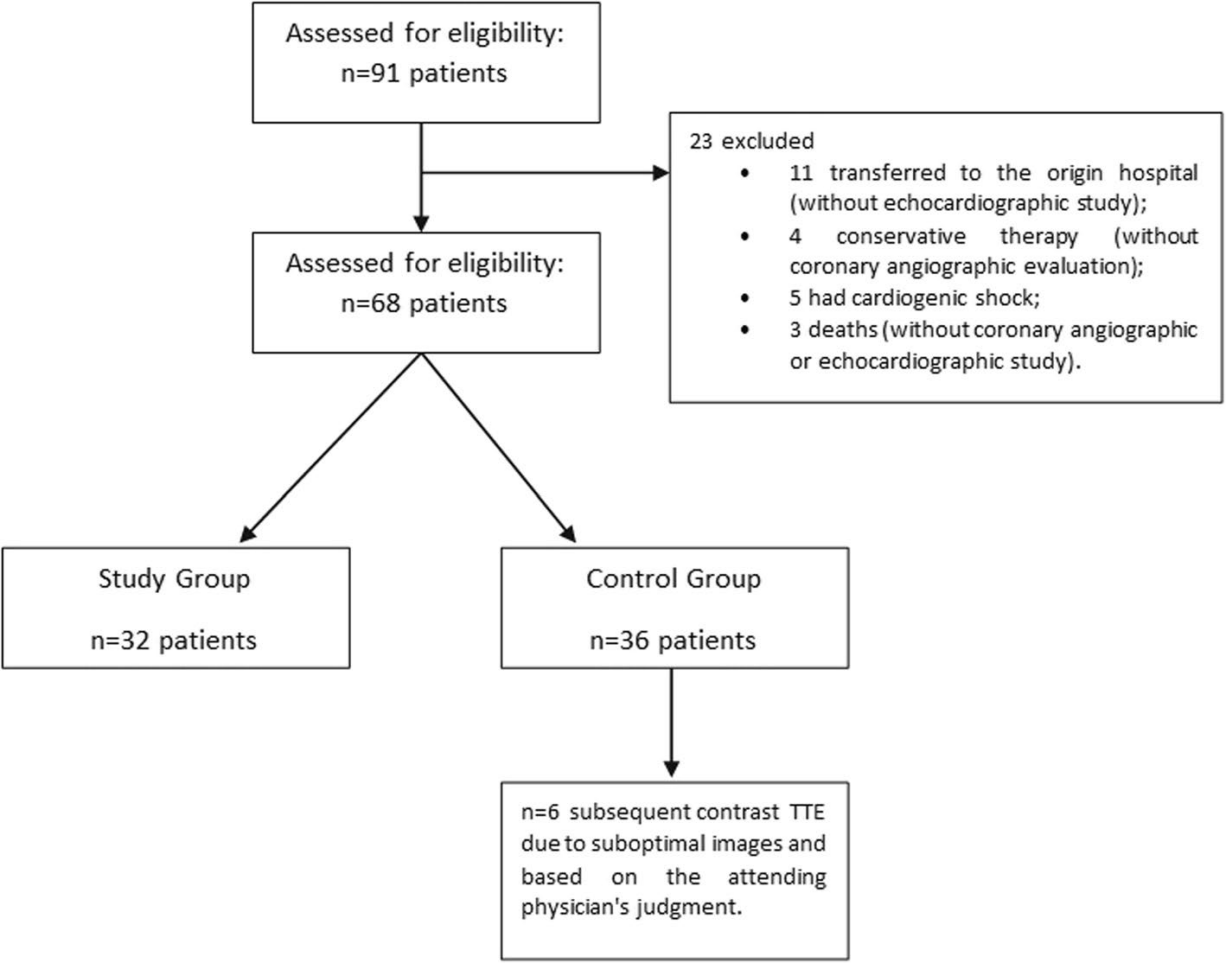
Study flowchart. TTE, transthoracic echocardiography

A prospective randomized controlled trial was conducted at a single center, including patients admitted to the Cardiac Intensive Care Unit of Centro Hospitalar de Tondela-Viseu (Viseu, Portugal) due to anterior STEMI between November 2021 and January 2023. The definition of anterior STEMI was in accordance with the fourth universal definition of myocardial infarction with ST-segment elevation by the European Society of Cardiology. The study design is presented in Fig. 1. The exclusion criteria were patients under 18 years old, who did not undergo echocardiographic or coronary angiographic evaluation, cardiogenic shock, and previously known thrombus or allergic contrast reaction.

The study group included all patients admitted on odd numbered days of the month that performed a contrast TTE, and the control group included patients admitted on even numbered days and were submitted to a conventional TTE as part of their initial diagnostic assessment. When doubts persisted, based on the attending physician's judgment, a subsequent contrast TTE could be performed on the control group, following the regular diagnostic approach in place. The scheduled date for performing both conventional and contrast TTE was within 7 days of admission, contingent upon clinical stability and the availability of the echocardiography laboratory.

For the contrast TTE procedure, the SonoVue ultrasound agent (Bracco) and the GE i9 echocardiograph machine (GE HealthCare) were utilized. The procedure was conducted by a team of four cardiologists specialized in ultrasound imaging, who were aware of the study protocol. The minimum amount of ultrasonographic contrast necessary for effective opacification of the left ventricle was employed.

Demographic and clinical information for each participant was collected by reviewing the electronic medical records, following the research protocol approved by our institution. This information included age, anthropometric data, cardiovascular risk factors, family history of cardiovascular disease or sudden death at a young age, history of cerebrovascular disease, history of atrial flutter/fibrillation, and bleeding history. During hospitalization, data related to the acute coronary event (including time interval from the onset of symptoms to diagnosis, time between the electrocardiogram and guidewire crossing, or fibrinolysis administration), catheterization details (culprit artery, stent implantation, and procedure time), treatment specifics (type of treatment, reperfusion status, deferred PCI after fibrinolysis/viability assessment, and thrombolysis in myocardial infarction [TIMI] final flow) and echocardiography findings (LV ejection fraction, LV dimensions, presence of anterior/apical aneurysm/pseudoaneurysm, occurrence of mechanic complications, presence LV thrombus) were collected.

Late presentation myocardial infarction was defined as more than 12 h after symptom onset of symptoms and admission. Echocardiographic LV function was assessed using Simpson biplane method. LV thrombus was defined as an echo-dense mass in the LV, distinct from the endocardium and adjacent to an area of hypokinetic or akinetic myocardium, with a complete absence of contrast uptake after injection of ultrasound contrast (when indicated). Mechanic complication was defined as rupture of the LV free wall, rupture of the interventricular septum, and acute mitral regurgitation due to papillary muscle necrosis.

The clinical and demographic characteristics were summarized using the median and interquartile range for continuous variables and relative frequencies for categorical variables. Statistical analysis was performed to compare the characteristics between the study group and the control group. Numerical variables were compared using the Mann–Whitney test, and categorical variables were compared using chi-square test or Fisher exact test, when appropriate. All statistical analyses were conducted using IBM SPSS ver. 20 (IBM Corp), with a significance level set at *P* < 0.05 to determine statistical significance.

## Results

A total of 68 patients were included in the study, with 32 patients assigned to the study group and 36 patients to the control group. Within the control group, six patients underwent a subsequent contrast TTE due to suboptimal images and based on the attending physician's judgment. Among these patients, three were overweight, one exhibited a large apical aneurysm, one displayed apical hypertrabeculation, and one had a suspected small thrombus identified through conventional TTE. None of the performed contrast studies were deemed inconclusive. The mean amount of ultrasonic contrast used was 1.0 ± 0.5 mL.

Demographic, clinical, and procedural data demonstrated similarity between the two groups (Table 1). Among the enrolled patients, the mean age was 66.1 ± 13.5 years, 55 (80.9%) were male, 14 (20.6%) had diabetes, 40 (58.8%) had arterial hypertension, 35 (51.5%) had dyslipidemia, and 34 (50.0%) had history of smoking (21 active smokers [30.9%] and 13 former smokers [19.1%]).

**Table 1:** Demographic and clinical characteristics (*n* = 68)

Characteristic	Study group (*n* = 32)	Control group (*n* = 36)	*P*-value
Male sex	26 (81.3)	29 (80.6)	0.94
Age (yr)	68.5 (53.8–77.5)	66.5 (53.3–79.5)	0.97
Hospitalization (day)	6 (5–10.5)	6.5 (5–8.8)	0.73
Body mass index (kg/m^2^)	25.7 (24.1–29.9)	26.8 (23.4–30.6)	0.53
Arterial hypertension	18 (56.3)	22 (61.1)	0.68
Diabetes	7 (21.9)	7 (19.4)	0.81
Dyslipidemia	17 (53.1)	18 (50.0)	0.80
Smoking status			0.51
Non-smoking	14 (43.8)	20 (55.6)	
Active smoking	12 (37.5)	9 (25.0)	
Previous smoker	6 (18.8)	7 (19.4)	
Alcohol abuse	3 (9.38)	7 (21.9)	0.24
Family history of cardiovascular disease in young age or sudden cardiac death	2 (6.3)	2 (5.6)	0.90
History of ischemic heart disease	7 (21.9)	4 (11.1)	0.23
Prior stroke	1 (3.1)	1 (2.8)	0.90
Chronic kidney disease	1 (3.3)	3 (8.3)	0.36
Chronic liver disease	2 (6.3)	1 (2.8)	0.49
Chronic obstructive pulmonary disease	2 (6.3)	2 (5.6)	0.90
Heart failure	0	1 (2.8)	0.34
Atrial fibrillation/flutter	3 (9.4)	1 (2.8)	0.25
Previous bleeding^a^	0	0	NA

Values are presented as number (%) or median (interquartile range Q1-Q3)

*NA* Not applicable

^a^Major bleeding was defined as clinically overt bleeding which was associated with any of the following: (1) a fall in hemoglobin level of 2 g/dL or more or documented transfusion of packed red blood cells or (2) involvement of a critical anatomical site (intracranial, spinal, ocular, pericardial, articular, intramuscular with compartment syndrome, retroperitoneal)

Table 2 outlines the distribution of coronary lesions categorized according to the American College of Cardiology/American Heart Association classification [12]. The majority of the cases presented involvement of the anterior descending artery as the culprit lesion, and only one patient in the control group exhibited a lesion in the second diagonal artery. ACC/AHA, American College of Cardiology/American Heart Association

**Table 2:** Coronary lesions distribution according to the ACC/AHA classification (*n* = 68)

Variable	No. of patients (%)		*P*-value
	Study group (*n* = 32)	Control group (*n* = 36)	
**Anterior descending artery**	**32 (100)**	**35 (97.2)**	> 0.99
- Ostial anterior descending artery	2 (6.3)	5 (13.9)	
- Proximal anterior descending artery	12 (37.5)	12 (33.3)	
- Mid anterior descending artery	9 (28.1)	13 (36.1)	
- Distal anterior descending artery	9 (28.1)	5 (13.9)	
**Second diagonal artery**	**0**	**1 (2.8)**	
- Proximal second diagonal artery	0	1 (2.8)	

Late presentation occurred in 4 patients (12.5%) in the study group and 5 patients (13.9%) in the study and control group. Fibrinolysis was administered in 18.8% (*n* = 6) of the patients in the study group and 33.3% (*n* = 12) in the control group. Across the entire patient cohort, a successful reperfusion was observed in 63 cases (92.6%). The detailed analysis concerning reperfusion status and the selected treatment modality for each group is presented in Table 3, while the reperfusion timelines are described in Table 4. These data uphold the similarity between the two groups. Percentages may not total 100 due to rounding

**Table 3:** Reperfusion status and type of treatment (*n* = 68)

Variable	No. of patients (%)		*P*-value
	Study group (*n* = 32)	Control group (*n* = 36)	
Successful reperfusion			0.66
Yes	29 (90.6)	34 (94.4)	
No	3 (9.4)	2 (5.6)	
Reperfusion method			0.45
Primary PCI	23 (71.9)	19 (52.8)	
Rescue PCI	4 (12.5)	8 (22.2)	
Deferred PCI after fibrinolysis	2 (6.3)	5 (13.9)	
PCI after viability assessment	0	1 (2.8)	
Cardiac surgery	0	1 (2.8)	
Medical treatment	3 (9.4)	2 (5.6)	
Fibrinolysis treatment	6 (18.8)	12 (33.3)	0.17
With reperfusion criteria	2 (33.0)	6 (50.0)	0.64

*PCI* Percutaneous coronary intervention

Values are presented as median (interquartile range) or number (%)

**Table 4:** Time intervals between symptoms onset to diagnosis and regarding ECG to reperfusion in different strategies (*n* = 68)

Variable	Study group (*n* = 32)	Control group (*n* = 36)	*P*-value
Time from symptoms to diagnosis (min)	156 (100–225)	166 (78–250)	0.90
Time from ECG to guidewire crossing (min)			
Primary PCI	123 (83–199)	112 (92–233)	0.38
Rescue PCI	195 (118.8–228)	193 (140.5–210.3)	> 0.99
Time from ECG to needle (fibrinolysis with reperfusion criteria) (min)	25.5 (-)	12.5 (10–31.5)	> 0.99
Procedure time (if PCI) (min)	53 (47–59)	54 (43–60)	0.77
Late presentation myocardial infarction	4 (12.5)	5 (13.9)	0.87

*ECG* Electrocardiogram, *PCI* Percutaneous coronary intervention

The median interval from the admission to the echocardiographic study was 4 days (interquartile range Q1(3)-Q3(5) for control group and Q1(3)-Q3(5.5) for study group) for both groups. No complications were observed with the use of ultrasound contrast. Echocardiographic characteristics and specific diagnoses are provided in Table 5. A significant statistical difference was noted between the two groups regarding the prevalence of anterior/apical aneurysms, with a higher incidence in the study group compared to the control group (46.9% vs, 22.2%, *P* = 0.03). However, there was no significant statistical difference found in the diagnosis of thrombus between the two groups (*P* = 0.24), with thrombus detection occurring in eight cases (25.0%) in the study group and five cases (13.9%) in the control group.

**Table 5:** Echocardiographic characteristics and specific diagnosis (*n* = 68)

Variable	Study group (*n* = 32)	Control group (*n* = 36)	*P*-value
From event to echocardiography (day)	4 (3–5.5)	4 (3–5)	0.89
Left ventricular ejection fraction (%)	40 (35–44.5)	43 (37–48.8)	0.65
LV indexed diastolic volume (cm^3^/m^2^)	54.5 (45–68)	51 (46–63)	0.56
Anterior/apical aneurysm	15 (46.9)	8 (22.2)	0.03^*^
Pseudoaneurysm	0	0	NA
Left ventricular thrombus	8 (25.0)	5 (13.9)	0.24
Mechanic complications^a^	1 (3.1)	0	0.29

Values are presented as median (interquartile range) or number (%)

*NA* Not applicable

^a^Defined as rupture of the left ventricular free wall, rupture of the interventricular septum, and acute mitral regurgitation due to papillary muscle necrosis

^*^P < 0.05

Excluding the six patients from the control group who underwent a subsequent contrast TTE, the thrombus detection rate was 10% (*n* = 3) among the remaining 30 patients in the control group. Even when excluding these cases, there was no statistically significant difference in thrombus detection rates between the control and study groups (*P* = 0.12).

The overall percentage of thrombus detection was 19% (*n* = 13). Of those, only 15.5% had a final non-TIMI 3 flow, with no statistically significant difference compared to the group without thrombus (Additional File 1).

The median length of hospital stay was 6 days for both groups. Only one case of mechanical complication, specifically interventricular septum rupture, was observed in the study group. The overall mortality rate was 1.5% (*n* = 1), corresponding to a patient who died due to nosocomial pneumonia.

## Discussion

LV thrombus formation is a serious complication that can occur following AMI, significantly increasing the risk of embolic complications and mortality [1, 2, 13]. This condition is associated with higher intrahospital and overall mortality rates, as well as heart failure with reduced LV function and ventricular dysrhythmias [3, 5]. Several factors contribute to LV thrombus development in this scenario, including blood stasis, which is more pronounced in cases of apical/anterior aneurysms, large infarct area, and reduced LV ejection fraction. Additionally, subendocardial damage resulting from prolonged ischemia, and a hypercoagulable state induced by the inflammatory response also contribute to thrombus formation [7, 14–17]. Accurate detection of LV thrombus is critical for initiating appropriate anticoagulant treatment and improving clinical outcomes [9, 10].

The standard imaging technique for detecting LV thrombus is two-dimensional TTE. However, TTE has certain limitations, including difficult echocardiographic windows, artifacts, and potential misinterpretation of structures within the LV, such as trabeculations or chordae [3, 5, 10]. To overcome these limitations, the incorporation of ultrasound contrast agents during TTE has been proposed to enhance the accuracy of LV thrombus detection [1, 18, 19]. Furthermore, ultrasound contrast agents have demonstrated a favorable safety profile [10, 11, 20]. The administration of these agents is contraindicated only in patients with known or suspected significant intracardiac cardiac shunting or a known hypersensitivity. Adverse events associated with contrast agents are rare, occurring in approximately one in 1,000 to 10,000 patients [10]. When adverse events do occur, they are generally mild, such as headache, nausea, dizziness, taste disturbances, paraesthesia, chest discomfort, or reactions at the injection site. These events are typically transient and do not require any specific treatment beyond providing reassurance to the patients [10, 11].

Our analysis revealed no substantial differences in demographic and clinical characteristics between the study and control groups, suggesting a reasonable baseline comparability. Furthermore, there were no noteworthy differences in the time since symptoms onset to diagnosis, rate of evolved myocardial infarction, distribution of coronary lesion, reperfusion status, reperfusion timings, or the selected treatment modalities between the study and control groups. The median interval from admission to the echocardiographic study, as well as LV indexed diastolic volume and ejection fraction, were similar in both cohorts. The significance of these parameters lies in their potential to influence critical aspects, such as the extent of myocardial damage, the size of the infarct area, the development of aneurysm and the reduction of LV ejection fraction. All these factors collectively contribute to the environment necessary for thrombus formation. For instance, timely and successful reperfusion, with promptly restore of the blood flow, prevents prolonged ischemia-induced damage, mitigating the physiopathological changes that lead to thrombus formation.

In the contemporary era, the incidence of LV thrombus following AMI has decreased to a range of 5% to 15% due to the widespread use of rapid mechanical reperfusion and potent antiplatelet and antithrombotic agents. The reported incidence varies depending on the patient population, screening protocols, and diagnostic test sensitivity, with a higher incidence observed in anterior-territory STEMI cases [1, 2, 7, 9, 21]. A meta-analysis that included more than 2,000 patients described an incidence of LV thrombus up to 12.2% in patients with anterior STEMI, and 19.2% in those with anterior STEMI and reduced LV ejection fraction (under 50%) detected by cardiac magnetic resonance [22]. The elevated rate of thrombus detection in our cohort (19%) could be linked to the substantial proportion of cases displaying reduced LV ejection fraction, with median values below 45% in both study and control groups.

In cases of anterior STEMI, the use of ultrasound contrast agents may be recommended to improve the accuracy of LV thrombus diagnosis [1, 18, 19]. However, in our study, despite a higher thrombus detection rate in the study group, the difference was not statistically significant. This suggests that the immediate diagnosis of LV thrombi during the acute phase of anterior STEM in our population was not notably influenced by the use of ultrasound contrast agents. However, the small number of patients in our study limits the generalizability of the findings. Therefore, the results should be interpreted cautiously. The authors consider that it might be reasonable to reserve contrast agents for cases with suboptimal imaging that impedes the adequate differentiation of the endocardium or with conditions that increase the risk of thrombus formation, such as apical/anterior aneurysms, large infarct area, and reduced LV ejection fraction.

Interestingly, the study group revealed a higher prevalence of anterior/apical aneurysms compared to the control group. This highlights the potential utility of ultrasound contrast in detecting and characterizing aneurysms in anterior STEMI. Apical aneurysms are recognized risk factors for subsequent intracavitary thrombus development, warranting intensified follow-up [14–17].

Therefore, the new findings of the current study highlight the enhanced identification of apical/anterior LV aneurysms through the systematic use of contrast TTE during the acute phase of anterior STEMI. Since these aneurysms are risk factors for thrombus formation, our results suggest a potential role for ultrasound contrast agents in refining risk stratification and surveillance strategies.

### Limitations

Interpreting our study’s results requires careful consideration of its limitations. Firstly, the sample size was relatively small, potentially limiting our ability to detect subtle differences between the study and control groups due to insufficient statistical power. Increasing the sample size would enhance the solidity of the conclusions pertaining to the influence of ultrasound contrast during the acute phase of anterior STEMI. Nevertheless, even though our results imply that conventional TTE alone might satisfactorily diagnose LV thrombus in such cases, a larger sample size would probably reveal more pronounced outcomes, considering the higher absolute percentage of identified LV thrombi in the study group.

Additionally, our study was exclusively conducted at a single center, which may limit the applicability of our findings to other healthcare environments. Variations in patient demographics and management strategies among institutions can impact the external validity of the results. Therefore, it is important to exercise caution when extrapolating these findings to a broader patient population.

Our center experiences a significant number of cases involving fibrinolysis therapy. This is attributed to our status of PCI center that serves a vast territorial area. Moreover, we provide support to two non-PCI centers located more than 120 min away. This might introduce variations in the clinical characteristics and management strategies, potentially influencing the generalizability of our findings to centers with different reperfusion rates and patient demographics.

This was not a blinded study, which may introduce potential bias. In the context of this research, the operators conducting the echocardiographic exams searching for thrombi could have influenced their focus and, consequently, heightened the identification rates. Other factors, such as the specific ultrasound equipment utilized, could have also influenced our results, as different machines can differ in image quality and capabilities. Moreover, the relatively short time gap between AMI and the TTE performance could potentially affect the detectability of the LV thrombi, considering that apical thrombus can also occur 7 to 14 days after AMI, a period that may not have been captured in our study's acute-phase focus. Additionally, it is important to note that some patients may not have been within this window due to shorter hospitalization periods.

Lastly, our study exclusively concentrated on the acute phase of the disease. Long-term follow-up data were inaccessible, which hindered our capacity to evaluate potential extended repercussions and results linked to LV thrombus diagnoses after hospital discharge, as well as the influence of anticoagulation therapy on overall outcomes.

To obtain more meaningful results, further research using larger, multicenter studies with long-term follow-up is necessary to validate our findings and determine the clinical benefits and cost-effectiveness of incorporating ultrasound contrast into routine management for patients with anterior STEMI.

## Conclusions

The complexity of thrombus formation following anterior STEMI, emphasizes the multifactorial nature of this complication, including factors such as impaired LV function, large infarct size, and aneurysm formations. Accurate detection of LV thrombus is crucial for appropriate treatment and optimal clinical outcomes. The use of ultrasound contrast agents during TTE has the potential to enhance sensitivity, particularly in patients with anterior STEMI. Although the addition of ultrasound contrast did not significantly improve the immediate detection of LV thrombi in our population, the limited sample size may have affected the statistical power. However, our study revealed a potential role for ultrasound contrast in detecting apical aneurysms, which are known risk factors for subsequent intracavitary thrombus development. To obtain more meaningful results, further research using larger, multicenter studies with long-term follow-up is necessary.

### Supplementary Information

Additional file 1: Supplementary Table 1.

## Data Availability

Not applicable.

## Abbreviations

AMI: Acute myocardial infarction
LV: Left ventricular
PCI: Percutaneous coronary intervention
STEMI: ST-elevation myocardial infarction
TIMI: Thrombolysis in myocardial infarction
TTE: Transthoracic echocardiography
