# Supplementary material for: Left ventricular thrombus routine screening with contrast echocardiography in patients with anterior ST-elevation myocardial infarction: is it worth it?
Source: J Cardiovasc Imaging. 2024 Aug 5;32:21. doi: 10.1186/s44348-024-00027-0 (PMC11299253; doi:10.1186/s44348-024-00027-0)
Supplement: Supplementary file 1 — Additional file 1: Supplementary Table 1. [file 44348_2024_27_MOESM1_ESM.docx]

*Supplementary Table 1 – Comparison between the thrombus presence and TIMI flow (n – number; PCI – percutaneous coronary intervention; % - percentage)*

|  | **Thrombus (n=13)** | **No thrombus (n=55)** | **p-value** |
| --- | --- | --- | --- |
| TIMI III - n (%) | 11 (84.6) | 46 (83.6) | 1.00 |
| Non-TIMI III - n (%) | 2 (15.5) | 9 (16.4) |  |
